# Certifying Inexpressibility

**DOI:** 10.1007/978-3-030-71995-1_20

**Published:** 2021-03-23

**Authors:** Orna Kupferman, Salomon Sickert

**Affiliations:** 8 University of Oxford, Oxford, UK; 9 Sorbonne Université - LIP6, Paris, France; 10 School of Computer Science and Engineering, The Hebrew University, Jerusalem, Israel; 11 Technische Universität München, Munich, Germany

**Keywords:** Automata on infinite words, Expressive power, Games

## Abstract

Different classes of automata on infinite words have different expressive power. Deciding whether a given language $L \subseteq \varSigma ^\omega$ can be expressed by an automaton of a desired class can be reduced to deciding a game between Prover and Refuter: in each turn of the game, Refuter provides a letter in $\varSigma$, and Prover responds with an annotation of the current state of the run (for example, in the case of Büchi automata, whether the state is accepting or rejecting, and in the case of parity automata, what the color of the state is). Prover wins if the sequence of annotations she generates is correct: it is an accepting run iff the word generated by Refuter is in *L*. We show how a winning strategy for Refuter can serve as a simple and easy-to-understand certificate to inexpressibility, and how it induces additional forms of certificates. Our framework handles all classes of deterministic automata, including ones with structural restrictions like weak automata. In addition, it can be used for refuting *separation* of two languages by an automaton of the desired class, and for finding automata that *approximate* *L* and belong to the desired class.

## 1 Introduction

Finite *automata on infinite objects* were first introduced in the 60's, and were the key to the solution of several fundamental decision problems in mathematics and logic [8,33,41]. Today, automata on infinite objects are used for specification, verification, and synthesis of nonterminating systems. The automata-theoretic approach reduces questions about systems and their specifications to questions about automata [28,49], and is at the heart of many algorithms and tools. Industrial-strength property-specification languages such as the IEEE 1850

* The full version of this article is available from [27]. Orna Kupferman is supported in part by the Israel Science Foundation, grant No. 2357/19. Salomon Sickert is supported in part by the Deutsche Forschungsgemeinschaft (DFG) under project numbers 436811179 and 317422601 ("Verified Model Checkers"), and in part funded by the European Research Council (ERC) under the European Union's Horizon 2020 research and innovation programme under grant agreement No. 787367 (PaVeS).

Standard for Property Specification Language (PSL) [14] include regular expressions and/or automata, making specification and verification tools that are based on automata even more essential and popular.

A run $r$ of an automaton on infinite words is an infinite sequence of states, and acceptance is determined with respect to the set of states that $r$ visits infinitely often. For example, in *Büchi* automata, some of the states are designated as accepting states, denoted by $\alpha$, and a run is accepting iff it visits states from the accepting set $\alpha$ infinitely often [8]. Dually, in *co-Büchi* automata, a run is accepting if it visits the set $\alpha$ only finitely often. Then, in *parity* automata, the acceptance condition maps each state to a color in some set $C = \{j, \ldots, k\}$, for $j \in \{0, 1\}$ and some *index* $k \geq 0$, and a run is accepting if the maximal color it visits infinitely often is odd.

The different classes of automata have different *expressive power*. For example, while deterministic parity automata can recognize all $\omega$-regular languages, deterministic Büchi automata cannot [29]. We use DBW, DCW, and DPW to denote a deterministic Büchi, co-Büchi, and parity word automaton, respectively, or (this would be clear from the context) the set of languages recognizable by the automata in the corresponding class. There has been extensive research on expressiveness of automata on infinite words [48,20]. In particular, researchers have studied two natural expressiveness hierarchies induced by different classes of deterministic automata. The first hierarchy is the *Mostowski Hierarchy*, induced by the index of parity automata [35,50]. Formally, let DPW$[0, k]$ denote a DPW with $C = \{0, \ldots, k\}$, and similarly for DPW$[1, k]$ and $C = \{1, \ldots, k\}$. Clearly, DPW$[0, k] \subseteq$ DPW$[0, k + 1]$, and similarly DPW$[1, k] \subseteq$ DPW$[1, k+1]$. The hierarchy is infinite and strict. Moreover, DPW$[0, k]$ complements DPW$[1, k+1]$, and for every $k \geq 0$, there are languages $L_k$ and $L'_k$ such that $L_k \in$ DPW$[0, k] \setminus$ DPW$[1, k + 1]$ and $L'_k \in$ DPW$[1, k + 1] \setminus$ DPW$[0, k]$. At the bottom of this hierarchy, we have DBW and DCW. Indeed, DBW=DPW$[0, 1]$ and DCW=DPW$[1, 2]$.

While the Mostowski Hierarchy refines DPWs, the second hierarchy, which we term the *depth hierarchy*, refines deterministic *weak* automata (DWWs). Weak automata can be viewed as a special case of Büchi or co-Büchi automata in which every strongly connected component in the graph induced by the structure of the automaton is either contained in $\alpha$ or is disjoint from $\alpha$, where $\alpha$ is depending on the acceptance condition the set of accepting or rejecting states. The structure of weak automata captures the alternation between greatest and least fixed points in many temporal logics, and they were introduced in this context in [36]. DWWs have been used to represent vectors of real numbers [6], and they have many appealing theoretical and practical properties [32,21]. In terms of expressive power, DWW = DCW $\cap$ DBW.

The depth hierarchy is induced by the depth of alternation between accepting and rejecting components in DWWs. For this, we view a DWW as a DPW in which the colors visited along a run can only increase. Accordingly, each run eventually gets trapped in a single color, and is accepting iff this color is odd. We use DWW$[0, k]$ and DWW$[1, k]$ to denote weak-DPW$[0, k]$ and weak-

DPW[1, $k$], respectively. The picture obtained for the depth hierarchy is identical to that of the Mostowski hierarchy, with DWW[$j, k$] replacing DPW[$j, k$] [50]. At the bottom of the depth hierarchy we have *co-safety* and *safety* languages [2]. Indeed, co-safety languages are DWW[0, 1] and safety are DWW[1, 2].

Beyond the theoretical interest in expressiveness hierarchies, their study is motivated by the fact many algorithms, like synthesis and probabilistic model checking, need to operate on deterministic automata [5,3]. The lower the automata are in the expressiveness hierarchy, the simpler are algorithms for reasoning about them. Simplicity goes beyond complexity, which typically depends on the parity index [16], and involves important practical considerations like minimization and canonicity (exists only for DWWs [32]), circumvention of Safra's determinization [26], and symbolic implementations [47]. Of special interest is the characterization of DBWs. For example, it is shown in [25] that given a *linear temporal logic* formula $\psi$, there is an *alternation-free* $\mu$*-calculus* formula equivalent to $\forall\psi$ iff $\psi$ can be recognized by a DBW. Further research studies *typeness* for deterministic automata, examining the ability to define a weaker acceptance condition on top of a given automaton [19,21].

Our goal in this paper is to provide a simple and easy-to-understand explanation to inexpressibility results. The need to accompany results of decision procedures by an explanation (often termed "certificate") is not new, and includes certification of a "correct" decision of a model checker [24,44], reachability certificates in complex multi-agent systems [1], and explainable reactive synthesis [4]. To the best of our knowledge, our work is the first to provide certification to inexpressibility results.

The underlying idea is simple: Consider a language $L$ and a class $\gamma$ of deterministic automata. We consider a turn-based two-player game in which one player (Refuter) provides letters in $\Sigma$, and the second player (Prover) responds with letters from a set $A$ of annotations that describe states in a deterministic automaton. For example, when we consider a DBW, then $A = \{\text{ACC}, \text{REJ}\}$, and when we consider a DPW[0, $k$], then $A = \{0, \ldots, k\}$. Thus, during the interaction, Refuter generates a word $x \in \Sigma^\omega$ and Prover responds with a word $y \in A^\omega$. Prover wins if for all words $x \in \Sigma^\omega$, we have that $x \in L$ iff $y$ is accepting according to $\gamma$. Clearly, if there is a deterministic $\gamma$ automaton for $L$, then Prover can win by following its run on $x$. Dually, a finite-state winning strategy for Prover induces a deterministic $\gamma$ automaton for $L$. The game-based approach is not new, and has been used for deciding the membership of given $\omega$-regular languages in different classes of deterministic automata [26]. Further, the game-based formulation is used in descriptive set theory to classify sets into hierarchies, see for example [39, Chapters 4 and 5] for an introduction that focuses on $\omega$-regular languages. Our contribution is a study of strategies for Refuter. Indeed, since the above described game is determined [9] and the strategies are finite-state, Refuter has a winning strategy iff no deterministic $\gamma$ automaton for $L$ exists, and this winning strategy can serve as a certificate for inexpressibility.

*Example 1.* Consider the language $L_{\neg\infty a} \subseteq \{a, b\}^\omega$ of all words with only finitely many $a$'s. It is well known that $L$ cannot be recognized by a DBW [29]. In Fig-

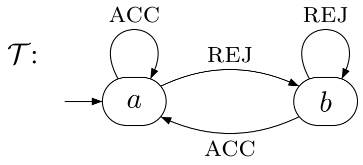

**Figure 1.** A refuter for DBW-recognizability of "only finitely many $a$'s".

ure 1 we describe what we believe to be the neatest proof of this fact. The figure describes a transducer $\mathcal{R}$ with inputs in {ACC,REJ} and outputs in $\{a, b\}$ – the winning strategy of Refuter in the above described game. The way to interpret $\mathcal{R}$ is as follows. In each round of the game, Prover tells Refuter whether the run of her DBW for $L_{\neg\infty a}$ is in an accepting or a rejecting state, and Refuter uses $\mathcal{R}$ in order to respond with the next letter in the input word. For example, if Prover starts with ACC, namely declaring that the initial state of her DBW is accepting, then Refuter responds with $a$, and if Prover continues with REJ, namely declaring that the state reachable with $a$ is rejecting, then Refuter responds with $b$. If Prover continues with REJ forever, then Prover continues with $b$ forever. Thus, together Prover and Refuter generate two words: $y \in \{\text{ACC}, \text{REJ}\}^\omega$ and $x \in \{a, b\}^\omega$. Prover wins whenever $x \in L_{\neg\infty a}$ iff $y$ contains infinitely many ACC's. If Prover indeed has a DBW for $L_{\neg\infty a}$, then she can follow its transition function and win the game. By following the refuter $\mathcal{R}$, however, Refuter can always fool Prover and generate a word $x$ such that $x \in L_{\neg\infty a}$ iff $y$ contains only finitely many ACC's. ■

We first define refuters for DBW-recognizability, and study their construction and size for languages given by deterministic or nondeterministic automata. Our refuters serve as a first inexpressibility certificate. We continue and argue that each DBW-refuter for a language $L$ induces three words $x \in \Sigma^*$ and $x_1, x_2 \in \Sigma^*$, such that $x \cdot (x_1 + x_2)^* \cdot x_1^\omega \subseteq L$ and $x \cdot (x_1^* \cdot x_2)^\omega \cap L = \emptyset$. The triple $\langle x, x_1, x_2 \rangle$ is an additional certificate for $L$ not being in DBW. Indeed, we show that a language $L$ is not in DBW iff it has a certificate as above. For example, the language $L_{\neg\infty a}$ has a certificate $\langle \epsilon, b, a \rangle$. In fact, we show that Landweber's proof for $L_{\neg\infty a}$ can be used as is for all languages not in DBW, with $x_1$ replacing $b$, $x_2$ replacing $a$, and adding $x$ as a prefix.

We then generalize our results on DBW-refutation and certification in two orthogonal directions. The first is an extension to richer classes of deterministic automata, in particular all classes in the two hierarchies discussed above, as well as all deterministic Emerson-Lei automata (DELWs) [17]. For the depth hierarchy, we add to the winning condition of the game a *structural restriction*. For example, in a weak automaton, Prover loses if the sequence $y \in A^\omega$ of annotations she generates includes infinitely many alternations between ACC and REJ. We show how structural restrictions can be easily expressed in our framework.

The second direction is an extension of the recognizability question to the questions of *separation* and *approximation*: We say that a language $L \subseteq \Sigma^\omega$ is a *separator* for two languages $L_1, L_2 \subseteq \Sigma^\omega$ if $L_1 \subseteq L$ and $L \cap L_2 = \emptyset$. Studies of separation include a search for regular separators of general languages [11], as well as separation of regular languages by weaker classes of languages, e.g., FO-definable languages [40] or piecewise testable languages [12]. In the context of $\omega$-regular languages, [2] presents an algorithm computing the smallest safety language containing a given language $L_1$, thus finding a safety separator for $L_1$ and $L_2$. As far as we know, besides this result there has been no systematic study of separation of $\omega$-regular languages by deterministic automata.

In addition to the interest in separators, we use them in the context of recognizability in two ways. First, a third type of certificate that we suggest for DBW-refutation of a language $L$ are "simple" languages $L_1$ and $L_2$ such that $L_1 \subseteq L$, $L \cap L_2 = \emptyset$, and $\langle L_1, L_2 \rangle$ are not DBW-separable. Second, we use separability in order to approximate languages that are not in DBW. Consider such a language $L \subseteq \Sigma^\omega$. A user may be willing to approximate $L$ in order to obtain DBW-recognizability. Specifically, we assume that there are languages $I_\downarrow \subseteq L$ and $I_\uparrow \subseteq \Sigma^\omega \setminus L$ of words that the user is willing to under- and over-approximate $L$ with. Thus, the user searches for a language that is a separator for $L \setminus I_\downarrow$ and $\Sigma^\omega \setminus (L \cup I_\uparrow)$. We study DBW-separability and DBW-approximation, namely separability and approximation by languages in DBW. In particular, we are interested in finding "small" approximating languages $I_\downarrow$ and $I_\uparrow$ with which $L$ has a DBW-approximation, and we show how certificates that refute DBW-separation can direct the search to for successful $I_\downarrow$ and $I_\uparrow$. Essentially, as in *counterexample guided abstraction-refinement* (CEGAR) for model checking [10], we use certificates for non-DBW-separability in order to suggest interesting *radius languages*. While in CEGAR the refined system excludes the counterexample, in our setting the approximation of $L$ excludes the certificate. As has been the case with recognizability, we extend our results to all classes of deterministic automata.

## 2 Preliminaries

### 2.1 Transducers and Realizability

Consider two finite alphabets $\Sigma$ and $A$. It is convenient to think about $\Sigma$ as the "main" alphabet, and about $A$ as an alphabet of annotations. For two words $x = x_0 \cdot x_1 \cdot x_2 \cdots \in \Sigma^\omega$ and $y = y_0 \cdot y_1 \cdot y_2 \cdots \in A^\omega$, we define $x \oplus y$ as the word in $(\Sigma \times A)^\omega$ obtained by merging $x$ and $y$. Thus, $x \oplus y = (x_0, y_0) \cdot (x_1, y_1) \cdot (x_2, y_2) \cdots$.

A $(\Sigma/A)$-*transducer* models a finite-state system that responds with letters in $A$ while interacting with an environment that generates letters in $\Sigma$. Formally, a $(\Sigma/A)$-transducer is $\mathcal{T} = \langle \Sigma, A, \iota, S, s_0, \rho, \tau \rangle$, where $\iota \in \{sys, env\}$ indicates who initiates the interaction – the system or the environment, $S$ is a set of states, $s_0 \in S$ is an initial state, $\rho : S \times \Sigma \to S$ is a transition function, and $\tau : S \to A$ is a labelling function on the states. Consider an input word $x = x_0 \cdot x_1 \cdot x_2 \cdots \in \Sigma^\omega$. The *run* of $\mathcal{T}$ on $x$ is the sequence $s_0, s_1, s_2 \ldots$ such that for all $j \geq 0$, we have that $s_{j+1} = \rho(s_j, x_j)$. The *annotation of $x$ by $\mathcal{T}$*, denoted $\mathcal{T}(x)$, depends on $\iota$. If $\iota = sys$, then $\mathcal{T}(x) = \tau(s_0) \cdot \tau(s_1) \cdot \tau(s_2) \cdots \in A^\omega$. Note that the first letter in $A$ is the output of $\mathcal{T}$ in $s_0$. This reflects the fact that the system initiates the interaction. If $\iota = env$, then $\mathcal{T}(x) = \tau(s_1) \cdot \tau(s_2) \cdot \tau(s_3) \cdots \in A^\omega$. Note that now, the output in $s_0$ is ignored, reflecting the fact that the environment initiates the interaction.

Consider a language $L \subseteq (\Sigma \times A)^\omega$. Let $comp(L)$ denote the complement of $L$. Thus, $comp(L) = (\Sigma \times A)^\omega \setminus L$. We say that a language $L \subseteq (\Sigma \times A)^\omega$ is *$(\Sigma/A)$-realizable by the system* if there is a $(\Sigma/A)$-transducer $\mathcal{T}$ with $\iota = sys$ such that for every word $x \in \Sigma^\omega$, we have that $x \oplus \mathcal{T}(x) \in L$. Then, $L$ is *$(A/\Sigma)$-realizable by the environment* if there is an $(A/\Sigma)$-transducer $\mathcal{T}$ with $i = env$ such that for every word $y \in A^\omega$, we have that $\mathcal{T}(y) \oplus y \in L$. When the language $L$ is regular, realizability reduces to deciding a game with a regular winning condition. Then, by determinacy of games and due to the existence of finite-memory winning strategies [9], we have the following.

**Proposition 1.** *For every $\omega$-regular language $L \subseteq (\Sigma \times A)^\omega$, exactly one of the following holds.*

1. *$L$ is $(\Sigma/A)$-realizable by the system.*
2. *$comp(L)$ is $(A/\Sigma)$-realizable by the environment.*

### 2.2 Automata

A *deterministic word automaton* over a finite alphabet $\Sigma$ is $\mathcal{A} = \langle \Sigma, Q, q_0, \delta, \alpha \rangle$, where $Q$ is a set of states, $q_0 \in Q$ is an initial state, $\delta : Q \times \Sigma \to Q$ is a transition function, and $\alpha$ is an acceptance condition. We extend $\delta$ to words in $\Sigma^*$ in the expected way, thus for $q \in Q$, $w \in \Sigma^*$, and letter $\sigma \in \Sigma$, we have that $\delta(q, \epsilon) = q$ and $\delta(q, w\sigma) = \delta(\delta(q, w), \sigma)$. A *run* of $\mathcal{A}$ on an infinite word $\sigma_0, \sigma_1, \dots \in \Sigma^\omega$ is the sequence of states $r = q_0, q_1, \dots$, where for every position $i \geq 0$, we have that $q_{i+1} = \delta(q_i, \sigma_i)$. We use $inf(r)$ to denote the set of states that $r$ visits infinitely often. Thus, $inf(r) = \{q : \ q_i = q \text{ for infinitely many } i \geq 0\}$.

The acceptance condition $\alpha$ refers to $inf(r)$ and determines whether the run $r$ is accepting. For example, in the *Büchi*, acceptance condition, we have that $\alpha \subseteq Q$, and a run is accepting iff it visits states in $\alpha$ infinitely often; that is, $\alpha \cap inf(r) \neq \emptyset$. Dually, in *co-Büchi*, $\alpha \subseteq Q$, and a run is accepting iff it visits states in $\alpha$ only finitely often; that is, $\alpha \cap inf(r) = \emptyset$. The language of $\mathcal{A}$, denoted $L(\mathcal{A})$, is then the set of words $w$ such that the run of $\mathcal{A}$ on $w$ is accepting.

A parity condition is $\alpha : Q \to \{0, \dots, k\}$, for $k \geq 0$, termed the *index* of $\alpha$. A run $r$ satisfies $\alpha$ iff the maximal color $i \in \{0, \dots, k\}$ such that $\alpha^{-1}(i) \cap inf(r) \neq \emptyset$ is odd. That is, $r$ is accepting iff the maximal color that $r$ visits infinitely often is odd. Then, a Rabin condition is $\alpha = \{\langle G_1, B_1 \rangle, \dots, \langle G_k, B_k \rangle\}$, with $G_i, B_i \subseteq Q$, for all $0 \leq i \leq k$. A run $r$ satisfies $\alpha$ iff there is $1 \leq i \leq k$ such that $inf(r) \cap G_i \neq \emptyset$ and $inf(r) \cap B_i = \emptyset$. Thus, there is a pair $\langle G_i, B_i \rangle$ such that $r$ visits states in $G_i$ infinitely often and visits states in $B_i$ only finitely often.

All the acceptance conditions above can be viewed as special cases of the *Emerson-Lei acceptance condition* (EL-condition, for short) [17], which we define below. Let $\mathbb{M}$ be a finite set of marks. Given an infinite sequence $\pi = M_0 \cdot M_1 \cdots \in (2^{\mathbb{M}})^\omega$ of subsets of marks, let $inf(\pi)$ be the set of marks that appear infinitely often in sets in $\pi$. Thus, $inf(\pi) = \{m \in \mathbb{M} :$ there exist infinitely many $i \geq 0$ such that $m \in M_i\}$. An EL-condition is a Boolean assertion over atoms in $\mathbb{M}$. For simplicity, we consider assertions in positive normal form, where negation is applied only to atoms. Intuitively, marks that appear positively should repeat infinitely often and marks that appear negatively should repeat only finitely often. Formally, a deterministic EL-automaton is $\mathcal{A} = \langle \Sigma, Q, q_0, \delta, \mathbb{M}, \tau, \theta \rangle$, where $\tau \colon Q \to 2^{\mathbb{M}}$ maps each state to a set of marks, and $\theta$ is an EL-condition over $\mathbb{M}$. A run $r$ of a $\mathcal{A}$ is accepting if $inf(\tau(r))$ satisfies $\theta$.

For example, a Büchi condition $\alpha \subseteq Q$ can be viewed as an EL-condition with $\mathbb{M} = \{\textsc{acc}\}$ and $\tau(q) = \{\textsc{acc}\}$ for $q \in \alpha$ and $\tau(q) = \emptyset$ for $q \notin \alpha$. Then, the assertion $\theta = \textsc{acc}$ is satisfied by sequences $\pi$ induced by runs $r$ with $inf(r) \cap \alpha \neq \emptyset$. Dually, the assertion $\theta = \neg\textsc{rej}$ with $\mathbb{M} = \{\textsc{rej}\}$ is satisfied by sequences $\pi$ induced by runs $r$ with $inf(r) \cap \alpha = \emptyset$, and thus corresponds to a co-Büchi condition. In the case of a parity condition $\alpha : Q \to \{0, \ldots, k\}$, it is not hard to see that $\alpha$ is equivalent to an EL-condition in which $\mathbb{M} = \{0, 1, \ldots, k\}$, for every state $q \in Q$, we have that $\tau(q) = \{\alpha(q)\}$, and $\theta$ expresses the parity condition. Lastly, a Rabin condition $\alpha = \{\langle G_1, B_1 \rangle, \ldots, \langle G_k, B_k \rangle\}$ is equivalent to an EL-condition with $\mathbb{M} = \{G_1, B_1, \ldots, G_k, B_k\}$ and $\tau(q) = \{m \in \mathbb{M} : q \in m\}$. Note that now, the mapping $\tau$ is not to singletons, and each state is marked by all sets in $\alpha$ in which it is a member. Then, $\theta = \bigvee_{1 \leq i \leq k}(G_i \wedge \neg B_i)$.

We use DBW, DCW, DPW, DRW, DELW to denote deterministic Büchi, co-Büchi, parity, Rabin, and EL word automata, respectively. For parity automata, we also use DPW$[0, k]$ and DPW$[1, k]$, for $k \geq 0$, to denote DPWs in which the colours are in $\{0, \ldots, k\}$ and $\{1, \ldots, k\}$, respectively. For Rabin automata, we use DRW$[k]$, for $k \geq 0$, to denote DRWs that have at most $k$ elements in $\alpha$. Finally, we use DELW$[\theta]$, to denote DELWs with EL-condition $\theta$. We sometimes use the above acronyms in order to refer to the set of languages that are recognizable by the corresponding class of automata. For example, we say that a language $L$ is in DBW if $L$ is *DBW-recognizable*, thus there is a DBW $\mathcal{A}$ such that $L = L(\mathcal{A})$. Note that DBW = DPW$[0, 1]$, DCW = DPW$[1, 2]$, and DRW$[1]$ = DPW$[0, 2]$. In fact, in terms of expressiveness, DRW$[k]$ = DPW$[0, 2k]$ [43,31].

Consider a directed graph $G = \langle V, E \rangle$. A *strongly connected set* of $G$ (SCS) is a set $C \subseteq V$ of vertices such that for every two vertices $v, v' \in C$, there is a path from $v$ to $v'$. An SCS $C$ is *maximal* if it cannot be extended to a larger SCS. Formally, for every nonempty $C' \subseteq V \setminus C$, we have that $C \cup C'$ is not an SCS. The maximal strongly connected sets are also termed *strongly connected components* (SCC). An automaton $\mathcal{A} = \langle \Sigma, Q, Q_0, \delta, \alpha \rangle$ induces a directed graph $G_{\mathcal{A}} = \langle Q, E \rangle$ in which $\langle q, q' \rangle \in E$ iff there is a letter $\sigma$ such that $q' \in \delta(q, \sigma)$. When we talk about the SCSs and SCCs of $\mathcal{A}$, we refer to those of $G_{\mathcal{A}}$. Consider a run $r$ of an automaton $\mathcal{A}$. It is not hard to see that the set $inf(r)$ is an SCS. Indeed, since every two states $q$ and $q'$ in $inf(r)$ are visited infinitely often, the state $q'$ must be reachable from $q$.

## 3 Refuting DBW-Recognizability

Let $A = \{\text{ACC}, \text{REJ}\}$. We use $\infty\text{ACC}$ to denote the subset $\{a_0 \cdot a_1 \cdot a_2 \cdots \in A^\omega :$ there are infinitely many $j \geq 0$ with $a_j = \text{ACC}\}$ and $\neg\infty\text{ACC} = comp(\infty\text{ACC}) = \{a_0 \cdot a_1 \cdot a_2 \cdots \in A^\omega :$ there are only finitely many $j \geq 0$ with $a_j = \text{ACC}\}$.

A DBW $\mathcal{A} = \langle \Sigma, Q, q_0, \delta, \alpha \rangle$ can be viewed as a $(\Sigma/A)$-transducer $\mathcal{T}_\mathcal{A} = \langle \Sigma, A, sys, Q, q_0, \delta, \tau \rangle$, where for every state $q \in Q$, we have that $\tau(q) = \text{ACC}$ if $q \in \alpha$, and $\tau(q) = \text{REJ}$ otherwise. Then, for every word $x \in \Sigma^\omega$, we have that $x \in L(\mathcal{A})$ iff $\mathcal{T}_\mathcal{A}(x) \in \infty\text{ACC}$.

For a language $L \subseteq \Sigma^\omega$, we define the language $\text{DBW}(L) \subseteq (\Sigma \times A)^\omega$ of words with correct annotations. Thus,

$$\text{DBW}(L) = \{x \oplus y : x \in L \text{ iff } y \in \infty\text{ACC}\}.$$

Note that $comp(\text{DBW}(L))$ is the language

$$\text{NoDBW}(L) = \{x \oplus y : (x \in L \text{ and } y \notin \infty\text{ACC}) \text{ or } (x \notin L \text{ and } y \in \infty\text{ACC})\}.$$

A *DBW-refuter for* $L$ is an $(A/\Sigma)$-transducer with $\iota = env$ realizing $\text{NoDBW}(L)$.

*Example 2.* For every language $R \subseteq \Sigma^*$ of finite words, the language $R^\omega \subseteq \Sigma^\omega$ consists of infinite concatenations of words in $R$. It was recently shown that $R^\omega$ may not be in DBW [30]. The language used in [30] is $R = \$ + (0 \cdot \{0, 1, \$\}^* \cdot 1)$. In Figure 2 below we describe a DBW-refuter for $R^\omega$.

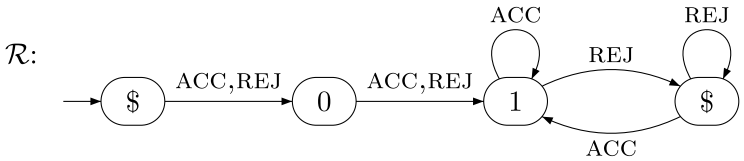

**Figure 2.** A DBW-refuter for $(\$ + (0 \cdot \{0, 1, \$\}^* \cdot 1))^\omega$.

Following $\mathcal{R}$, Refuter starts by generating a prefix $0 \cdot 1$ and then responds to ACC with 1 and responds with \$ to REJ. Accordingly, if Prover generates a rejecting run, Prover generates a word in $0 \cdot 1 \cdot (1 + \$)^* \cdot \$^\omega$, which is in $R^\omega$. Also, if Prover generates an accepting run, Prover generates a word in $0 \cdot 1 \cdot (1^+ \cdot \$^*)^\omega$, which has a single 0 and infinitely many 1's, and is therefore not in $R^\omega$. ■

By Proposition 1, we have the following.

**Proposition 2.** *Consider a language* $L \subseteq \Sigma^\omega$*. Let* $A = \{\text{ACC}, \text{REJ}\}$*. Exactly one of the following holds:*

- *$L$ is in DBW, in which case the language* $\text{DBW}(L)$ *is $(\Sigma/A)$-realizable by the system, and a finite-memory winning strategy for the system induces a DBW for $L$.*
- *$L$ is not in DBW, in which case the language* $\text{NoDBW}(L)$ *is $(A/\Sigma)$-realizable by the environment, and a finite-memory winning strategy for the environment induces a DBW-refuter for $L$.*

### 3.1 Complexity

In this section we analyze the size of refuters. We start with the case where the language $L$ is given by a DPW.

**Theorem 1.** *Consider a DPW $\mathcal{A}$ with $n$ states. Let $L = L(\mathcal{A})$. One of the following holds.*

1. *There is a DBW for $L$ with $n$ states.*
2. *There is a DBW-refuter for $L$ with $2n$ states.*

*Proof.* If $L$ is in DBW, then, as DPWs are Büchi type [19], a DBW for $L$ can be defined on top of the structure of $\mathcal{A}$, and so it has $n$ states. If $L$ is not in DBW, then by Proposition 2, there is a DBW-refuter for $L$, namely a $(\{\text{ACC}, \text{REJ}\}/\Sigma)$-transducer that realizes NoDBW$(L)$. We show we can define a DRW $\mathcal{U}$ with $2n$ states for NoDBW$(L)$. The result then follows from the fact a realizable DRW is realized by a transducer of the same size as the DRW [15].

We construct $\mathcal{U}$ by taking the union of the acceptance conditions of a DRW $\mathcal{U}_1$ for $\{x \oplus y : x \in L$ and $y \notin \infty\text{ACC}\}$ and a DRW $\mathcal{U}_2$ for $\{x \oplus y : x \notin L$ and $y \in \infty\text{ACC}\}$. We obtain both DRWs by taking the product of $\mathcal{A}$, extended to the alphabet $\Sigma \times \{\text{ACC}, \text{REJ}\}$, with a 2-state automaton for $\infty\text{ACC}$, again extended to the alphabet $\Sigma \times \{\text{ACC}, \text{REJ}\}$.

We describe the construction in detail. Let $\mathcal{A} = \langle \Sigma, Q, q_0, \delta, \alpha \rangle$. Then, the state space of $\mathcal{U}_1$ is $Q \times \{\text{ACC}, \text{REJ}\}$ and its transition on a letter $\langle \sigma, a \rangle$ follows $\delta$ when it reads $\sigma$, with $a$ determining whether $\mathcal{U}_1$ moves to the ACC or REJ copy. Let $\alpha_1$ be the Rabin condition equivalent to $\alpha$. We obtain the acceptance condition of $\mathcal{U}_1$ by replacing each pair $\langle G, B \rangle$ in $\alpha_1$ by $\langle G \times \{\text{REJ}\}, B \times \{\text{REJ}\} \cup Q \times \{\text{ACC}\} \rangle$. It is not hard to see that a run of $\mathcal{U}_1$ satisfies the latter pair iff its projection on $Q$ satisfies the pair $\langle G, B \rangle$ and its projection on $\{\text{ACC}, \text{REJ}\}$ has only finitely many ACC. The construction of $\mathcal{U}_2$ is similar, with $\alpha_2$ being a Rabin condition that complements $\alpha$, and then replacing each pair $\langle G, B \rangle$ in $\alpha_2$ by $\langle G \times \{\text{ACC}\}, B \times \{\text{ACC}, \text{REJ}\}) \rangle$. Since $\mathcal{U}_1$ and $\mathcal{U}_2$ have the same state space, and we only have to take the union of the pairs in their acceptance conditions, the $2n$ bound follows. □

Now, when $L$ is given by an NBW, an exponential bound follows from the exponential blow up in determinization [42]. If we are also given an NBW for $comp(L)$, the complexity can be tightened. Formally, we have the following.

**Theorem 2.** *Given NBWs with $n$ and $m$ states, for $L$ and $comp(L)$, respectively, one of the following holds.*

1. *There is a DBW for $L$ with $\min\{(1.65n)^n, 3^m\}$ states.*
2. *There is a DBW-refuter for $L$ with $\min\{2 \cdot (1.65n)^n, 2 \cdot (1.65m)^m\}$ states.*

*Proof.* If $L$ is in DBW, then a DBW for $L$ can be defined on top of a DPW for $L$, which has at most $(1.65n)^n$ states [45], or by dualizing a DCW for $comp(L)$. Since the translation of an NBW with $m$ states to a DCW, when it exists, results in a DCW with $3^m$ states [7], we are done. If $L$ is not in DBW, then we proceed as in the proof of Theorem 1, defining $\mathcal{U}$ on the top of a DPW for either $L$ or $comp(L)$. □

### 3.2 Certifying DBW-Refutation

Consider a DBW-refuter $\mathcal{R} = \langle\{\text{ACC}, \text{REJ}\}, \Sigma, env, S, s_0, \rho, \tau\rangle$. We say that a path $s_0, \ldots, s_m$ in $\mathcal{R}$ is an $\text{REJ}^+$-path if it contains at least one transition and all the transitions along it are labeled by REJ; thus, for all $0 \leq j < m$, we have that $s_{j+1} = \rho(s_j, \text{REJ})$. Then, a path $s_0, \ldots, s_m$ in $\mathcal{R}$ is an ACC-path if it contains at least one transition and its first transition is labeled by ACC. Thus, $s_1 = \rho(s_0, \text{ACC})$.

**Lemma 1.** *Consider a DBW-refuter $\mathcal{R} = \langle\{\text{ACC}, \text{REJ}\}, \Sigma, env, S, s_0, \rho, \tau\rangle$. Then there exists a state $s \in S$, a (possibly empty) path $p = s_0, s_1, \ldots s_m$, a $\text{REJ}^+$-cycle $p_1 = s^1_0, s^1_1 \ldots s^1_{m_1}$, and an ACC-cycle $p_2 = s^2_0, s^2_1 \ldots s^2_{m_2}$, such that $s_m = s^1_0 = s^1_{m_1} = s^2_0 = s^2_{m_2} = s$.*

*Proof.* Let $s_i \in S$ be a reachable state that belongs to an ergodic component in the graph of $\mathcal{R}$ (that is, $s_i \in C$, for a set $C$ of strongly connected states that can reach only states in $C$). Since $\mathcal{R}$ is responsive, in the sense it can read in each round both ACC and REJ, we can read from $s_i$ the input sequence $\text{REJ}^\omega$. Hence, $\mathcal{R}$ has a $\text{REJ}^+$-path $s_i, \ldots, s_l, \ldots, s_k$ with $s_l = s_k$, for $l < k$. It is easy to see that the claim holds with $s = s_l$. In particular, since $\mathcal{R}$ is responsive and $C$ is strongly connected, there exists an ACC-cycle from $s_l$ to itself. □

**Theorem 3.** *An $\omega$-regular language $L$ is not in DBW iff there exist three finite words $x \in \Sigma^*$ and $x_1, x_2 \in \Sigma^+$, such that $x \cdot (x_1 + x_2)^* \cdot x_1^\omega \subseteq L$ and $x \cdot (x_1^* \cdot x_2)^\omega \cap L = \emptyset$.*

*Proof.* Assume first that $L$ is not in DBW. Then, by Theorem 2, there exists a DBW-refuter $\mathcal{R}$ for it. Let $p = s_0, s_1, \ldots s_m$, $p_1 = s^1_0, s^1_1, \ldots, s^1_{m_1}$, and $p_2 = s^2_0, s^2_1, \ldots, s^2_{m_2}$, be the path, $\text{REJ}^+$-cycle, and ACC-cycle that are guaranteed to exist by Lemma 1. Let $x, x_1$, and $x_2$ be the outputs that $\mathcal{R}$ generates along them. Formally, $x = \tau(s_1)\cdot\tau(s_2)\cdots\tau(s_m)$, $x_1 = \tau(s^1_1)\cdot\tau(s^1_2)\cdots\tau(s^1_{m_1})$, and $x_2 = \tau(s^2_1) \cdot \tau(s^2_1) \cdots \tau(s^2_{m_2})$. Note that as the environment initiates the interaction, the first letter in the words $x$, $x_1$, and $x_2$, are the outputs in the second states in $p$, $p_1$, and $p_2$. The final step, i.e., that $x, x_1$, and $x_2$ satisfy the two conditions of the theorem, can be found in the full version of this article [27].

For the other direction, we adjust Landweber's proof [29] for the non-DBW-recognizability of $\neg\infty a$ to $L$. Essentially, $\neg\infty a$ can be viewed as a special case of $x\cdot(x_1+x_2)^*\cdot x_1^\omega$, with $x = \epsilon$, $x_1 = b$, and $x_2 = a$. Assume by way of contradiction that there is a DBW $\mathcal{A}$ with $L(\mathcal{A}) = L$. Let $\mathcal{A} = \langle\Sigma, Q, q_0, \delta, \alpha\rangle$. Consider the infinite word $w_0 = x \cdot x_1^\omega$. Since $w_0 \in x \cdot (x_1 + x_2)^* \cdot x_1^\omega$, and so $w \in L$, the run of $\mathcal{A}$ on $w_0$ is accepting. Thus, there is $i_1 \geq 0$ such that $\mathcal{A}$ visits $\alpha$ when it reads the $x_1$ suffix of $x \cdot x_1^{i_1}$. Consider now the infinite word $w_1 = x \cdot x_1^{i_1} \cdot x_2 \cdot x_1^\omega$. Since $w_1$ is also in $L$, the run of $\mathcal{A}$ on $w_1$ is accepting. Thus, there is $i_2 \geq 0$ such that $\mathcal{A}$ visits $\alpha$ when it reads the $x_1$ suffix of $x \cdot x_1^{i_1} \cdot x_2 \cdot x_1^{i_2}$. In a similar fashion we can continue to find indices $i_1, i_2, \ldots$ such for all $j \geq 1$, we have that $\mathcal{A}$ visits $\alpha$ when it reads the $x_1$ suffix of $x \cdot x_1^{i_1} \cdot x_2 \cdot x_1^{i_2} \cdot x_2 \cdots x_2 \cdot x_1^{i_j}$. Since $Q$ is finite, we can construct a word $w \in x \cdot (x_1^* \cdot x_2)^\omega$ that is accepted, but we assumed that

$x \cdot (x_1^* \cdot x_2)^\omega \cap L = \emptyset$, and thus we have reached a contradiction. The details of this step are given in [27]. □

We refer to a triple $\langle x, x_1, x_2 \rangle$ of words that satisfy the conditions in Theorem 3 as a *certificate* to the non-DBW-recognizability of $L$.

*Example 3.* In Example 2, we described a DBW-refuter for $L = (\$+(0\cdot\{0,1,\$\}^*\cdot 1))^\omega$. A certificate to its non-DBW-recognizability is $\langle x, x_1, x_2 \rangle$, with $x = 01$, $x_1 = \$$, and $x_2 = 1$. Indeed, $01 \cdot (\$ + 1)^* \cdot \$^\omega \subseteq L$ and $01 \cdot (\$^* \cdot 1)^\omega \cap L = \emptyset$. ■

Note that obtaining certificates according to the proof of Theorem 3 may not give us the shortest certificate. For example, for $L$ in Example 3, the proof would give us $x = 01\$$, $x_1 = \$$, and $x_2 = 1\$$, with $01\$ \cdot (\$ + 1\$)^* \cdot \$^\omega \subseteq L$ and $01\$ \cdot (\$^* \cdot 1\$)^\omega \cap L = \emptyset$. The problem of generating smallest certificates is related to the problem of finding smallest witnesses to DBW non-emptiness [22] and is harder. Formally, defining the length of a certificate $\langle x, x_1, x_2 \rangle$ as $|x|+|x_1|+|x_2|$, we have the following (see proof in [27]):

**Theorem 4.** *Consider a DPW $\mathcal{A}$ and a threshold $l \geq 1$. The problem of deciding whether there is a certificate of length at most $l$ for non-DBW-recognizability of $L(\mathcal{A})$ is NP-complete, for $l$ given in unary or binary.*

*Remark 1.* **[Relation with existing characterizations]** By [29], the language of a DPW $\mathcal{A} = \langle \Sigma, Q, q_0, \delta, \alpha \rangle$ is in DBW iff for every accepting SCS $C \subseteq Q$ and SCS $C' \supseteq C$, we have that $C'$ is accepting. The proof of Landweber relies on a complicated analysis of the structural properties of $\mathcal{A}$. As we elaborate in the full version [27], Theorem 3, which relies instead on determinacy of games, suggests an alternative proof. Similarly, [50] examines the structure of a deterministic Muller automaton, and Theorem 3 can be viewed as a special case of Lemma 14 there, with a proof based on the game setting.

■

Being an $(A/\Sigma)$-transducer, every DBW-refuter $\mathcal{R}$ is responsive and may generate many different words in $\Sigma^\omega$. Below we show that we can leave $\mathcal{R}$ responsive and yet let it generate only words induced by a certificate. Formally, we have the following.

**Lemma 2.** *Given a certificate $\langle x, x_1, x_2 \rangle$ to non-DBW-recognizability of a language $L \subseteq \Sigma^\omega$, we can define a refuter $\mathcal{R}$ for $L$ such that for every $y \in A^\omega$, if $y \models \infty\text{ACC}$, then $\mathcal{R}(y) \in x \cdot (x_1^* \cdot x_2)^\omega$, and if $y \models \neg\infty\text{ACC}$, then $\mathcal{R}(y) \in x \cdot (x_1 + x_2)^* \cdot x_1^\omega$.*

*Proof.* Intuitively, $\mathcal{R}$ first ignores the inputs and outputs $x$. It then repeatedly outputs either $x_1$ or $x_2$, according to the following policy: in the first iteration, $\mathcal{R}$ outputs $x_1$. If during the output of $x_1$ all inputs are REJ, then $\mathcal{R}$ outputs $x_1$ also in the next iteration. If an input ACC has been detected, thus the prover tries to accept the constructed word, the refuter outputs $x_2$ in the next iteration, again keeping track of an ACC input. If no ACC has been input, $\mathcal{R}$ switches back to outputting $x_1$. The formal definition of $\mathcal{R}$ can be found in [27]. □

By Theorem 3, every language not in DBW has a certificate $\langle x, x_1, x_2 \rangle$. As we argue below, these certificates are linear in the number of states of the refuters.

**Lemma 3.** *Let $\mathcal{R}$ be a DBW-refuter for $L \subseteq \Sigma^\omega$ with $n$ states. Then, $L$ has a certificate of the form $\langle x, x_1, x_2 \rangle$ such that $|x| + |x_1| + |x_2| \leq 2 \cdot n$.*

*Proof.* The paths $p$, $p_1$, and $p_2$ that induce $x$, $x_1$ and $x_2$ in the proof of Theorem 3 are simple, and so they are all of length at most $n$. Also, while these paths may share edges, we can define them so that each edge appears in at most two paths. Indeed, if an edge appears in all three path, we can shorten $p$. Hence, $|x| + |x_1| + |x_2| \leq 2 \cdot n$, and we are done. □

**Theorem 5.** *Consider a language $L \subseteq \Sigma^\omega$ not in DBW. The length of a certificate for the non-DBW-recognizability of $L$ is linear in a DPW for $L$ and is exponential in an NBW for $L$. These bounds are tight.*

*Proof.* The upper bounds follow from Theorem 1 and Lemma 3, and the exponential determinization of NBWs. The lower bound in the NBW case follows from the exponential lower bound on the size of shortest non-universality witnesses for non-deterministic finite word automata (NFW) [34]. We sketch the reduction: Let $L_n \subseteq \{0,1\}^*$ be a language such that the shortest witness for non-universality of $L_n$ is exponential in $n$, but $L_n$ has a polynomial sized NFW. We then define $L'_n = (L_n \cdot \$ \cdot (0^* \cdot 1)^\omega) + ((0+1)^* \cdot \$ \cdot (0+1)^* \cdot 0^\omega)$. It is clear that $L'_n$ has a NBW polynomial in $n$ and is not DBW-recognizable. Note that for every word $w \in L_n$, we have $w \cdot \$ \cdot (0+1)^\omega \subseteq L'_n$. Thus, in order to satisfy Theorem 3, every certificate $\langle x, x_1, x_2 \rangle$ needs to have $w \cdot \$$ as prefix of $x$, for some $w \notin L_n$. Hence, it is exponential in the size of the NBW. □

*Remark 2.* **[LTL]** When the language $L$ is given by an LTL formula $\varphi$, then DBW($\varphi$) $= \varphi \leftrightarrow$ **GF**Acc and thus an off-the-shelf LTL synthesis tool can be used to extract a DBW-refuter, if one exists. As for complexity, a doubly-exponential upper bound on the size of a DPW for NoDBW($L$), and then also on the size of DBW-refuters and certificates, follows from the double-exponential translation of LTL formulas to DPWs [49,42]. The length of certificates, however, and then, by Lemma 2, also the size of a minimal refuter, is related to the *diameter* of the DPW for NoDBW($L$), and we leave its tight bound open. ■

## 4 Separability and Approximations

Consider three languages $L_1, L_2, L \subseteq \Sigma^\omega$. We say that $L$ is a *separator* for $\langle L_1, L_2 \rangle$ if $L_1 \subseteq L$ and $L_2 \cap L = \emptyset$. We say that a pair of languages $\langle L_1, L_2 \rangle$ is *DBW-separable* iff there exists a language $L$ in DBW such that $L$ is a separator for $\langle L_1, L_2 \rangle$.

*Example 4.* Let $\Sigma = \{a, b\}$, $L_1 = (a+b)^* \cdot b^\omega$, and $L_2 = (a+b)^* \cdot a^\omega$. By [29], $L_1$ and $L_2$ are not in DBW. They are, however, DBW-separable. A witness for this is $L = (a^* \cdot b)^\omega$. Indeed, $L_1 \subseteq L$, $L \cap L_2 = \emptyset$, and $L$ is DBW-recognizable. ■

Consider a language $L \subseteq \Sigma^\omega$, and suppose we know that $L$ is not in DBW. A user may be willing to approximate $L$ in order to obtain DBW-recognizability. Specifically, we assume that there is a language $I \subseteq \Sigma^\omega$ of words that the user is *indifferent* about. Formally, the user is satisfied with a language in DBW that agrees with $L$ on all words that are not in $I$. Formally, we say that a language $L'$ *approximates* $L$ *with radius* $I$ if $L \setminus I \subseteq L' \subseteq L \cup I$. It is easy to see that, equivalently, $L'$ is a separator for $\langle L \setminus I, comp(L \cup I)\rangle$. Note that the above formulation embodies the case where the user has in mind different over- and under-approximation radiuses, thus separating $\langle L \setminus I_\downarrow, comp(L \cup I_\uparrow)\rangle$ for possibly different $I_\downarrow$ and $I_\uparrow$. Indeed, by defining $I = (I_\downarrow \cap L) \cup (I_\uparrow \setminus L)$, we get $\langle L \setminus I, comp(L \cup I)\rangle = \langle L \setminus I_\downarrow, comp(L) \setminus I_\uparrow\rangle$.

It follows that by studying DBW-separability, we also study DBW-approximation, namely approximation by a language that is in DBW, possibly with different over- and under-approximation radiuses.

*Remark 3.* [**From recognizability to separation**] It is easy to see that DBW-separability generalizes DBW-recognizability, as $L$ is in DBW iff $\langle L, comp(L)\rangle$ is DBW-separable. Given $L \subseteq \Sigma^\omega$, we say that a pair of languages $\langle L_1, L_2\rangle$ is a *no-DBW-witness* for $L$ if $L$ is a separator for $\langle L_1, L_2\rangle$ and $\langle L_1, L_2\rangle$ is not DBW-separable. Note that the latter indeed implies that $L$ is not in DBW.

A simple no-DBW witness for $L$ can be obtained as follows. Let $\mathcal{R}$ be a DBW refuter for $L$. Then, we define $L_1 = \{\mathcal{R}(y) : y \in \neg\infty\textsc{acc}\}$ and $L_2 = \{\mathcal{R}(y) : y \in \infty\textsc{acc}\}$. By the definition of DBW-refuters, we have $L_1 \subseteq L$ and $L_2 \cap L = \emptyset$, and so $\langle L_1, L_2\rangle$ is a no-DBW witness for $L$. It is simple, in the sense that when we describe $L_1$ and $L_2$ by a tree obtained by pruning the $\Sigma^*$-tree, then each node has at most two children – these that correspond to the responses of $\mathcal{R}$ to ACC and REJ. ∎

## 4.1 Refuting Separability

For a pair of languages $\langle L_1, L_2\rangle$, we define the language $\text{SepDBW}(L) \subseteq (\Sigma \times A)^\omega$ of words with correct annotations for separation. Thus,

$$\text{SepDBW}(L_1, L_2) = \{x \oplus y : (x \in L_1 \to y \in \infty\textsc{acc}) \wedge (x \in L_2 \to y \notin \infty\textsc{acc})\}.$$

Note that $comp(\text{SepDBW}(L_1, L_2))$ is then the language

$$\text{NoSepDBW}(L_1, L_2) = \{x \oplus y : (x \in L_1 \wedge y \notin \infty\textsc{acc}) \vee (x \in L_2 \wedge y \in \infty\textsc{acc})\}.$$

A *DBW-sep-refuter for* $\langle L_1, L_2\rangle$ is an $(A/\Sigma)$-transducer with $\iota = env$ that realizes $\text{NoSepDBW}(L_1, L_2)$.

*Example 5.* Consider the language $L_{\neg\infty a} = (a + b)^* \cdot b^\omega$, which is not DBW. Let $I = a^* \cdot b^\omega + b^* \cdot a^\omega$, thus we are indifferent about words with only one alternation between $a$ and $b$. In Figure 3 we describe a DBW-sep refuter for $\langle L_{\neg\infty a} \setminus I, comp(L_{\neg\infty a} \cup I)\rangle$. Note that the refuter generates only words in $a \cdot b \cdot a \cdot (a + b)^\omega$, whose intersection with $I$ is empty. Consequently, the refutation is similar to the DBW-refutation of $L_{\neg\infty a}$. ∎

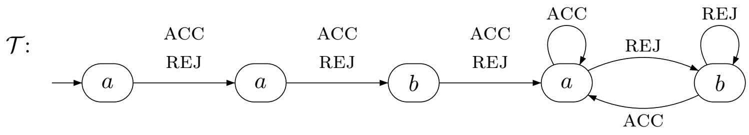

**Figure 3.** A DBW-sep refuter for $\langle L_{\neg\infty a} \setminus I, comp(L_{\neg\infty a} \cup I)\rangle$.

By Proposition 1, we have the following extension of Proposition 2.

**Proposition 3.** *Consider two languages $L_1, L_2 \subseteq \Sigma^\omega$. Let $A = \{\text{ACC}, \text{REJ}\}$. Exactly one of the following holds:*

- *$\langle L_1, L_2\rangle$ is DBW-separable, in which case the language* SepDBW$(L_1, L_2)$ *is $(\Sigma/A)$-realizable by the system, and a finite-memory winning strategy for the system induces a DBW for a language $L$ that separates $L_1$ and $L_2$.*
- *$\langle L_1, L_2\rangle$ is not DBW-separable, in which case the language* NoSepDBW$(L)$ *is $(A/\Sigma)$-realizable by the environment, and a finite-memory winning strategy for the environment induces a DBW-sep-refuter for $\langle L_1, L_2\rangle$.*

As for complexity, the construction of the game for SepDBW$(L_1, L_2)$ is similar to the one described in Theorem 1. Here, however, the input to the problem includes two DPWs. Also, the positive case, namely the construction of the separator does not follow from known results.

**Theorem 6.** *Consider DPWs $\mathcal{A}_1$ and $\mathcal{A}_2$ with $n_1$ and $n_2$ states, respectively. Let $L_1 = L(\mathcal{A}_1)$ and $L_2 = L(\mathcal{A}_2)$. One of the following holds.*

1. *There is a DBW $\mathcal{A}$ with $2 \cdot n_1 \cdot n_2$ states such that $L(\mathcal{A})$ DBW-separates $\langle L_1, L_2\rangle$.*
2. *There is a DBW-sep-refuter for $\langle L_1, L_2\rangle$ with $2 \cdot n_1 \cdot n_2$ states.*

*Proof.* We show that SepDBW$(L_1, L_2)$ and NoSepDBW$(L_1, L_2)$ can be recognised by DRWs with at most $2 \cdot n_1 \cdot n_2$ states. Then, by [15], we can construct a DBW or a DBW-sep-refuter with at most $2 \cdot n_1 \cdot n_2$ states. The construction is similar to the one described in the proof of Theorem 1. The only technical challenge is the fact SepDBW$(L_1, L_2)$ is defined as the intersection, rather than union, of two languages. For this, we observe that we can define SepDBW$(L_1, L_2)$ also as $\{x \oplus y : (y \in \infty\text{ACC}$ and $x \notin L_2)$ or $(y \notin \infty\text{ACC}$ and $x \notin L_1)\}$. With this formulation we then can reuse the union construction as seen in Theorem 1 to obtain DRWs with at most $2 \cdot n_1 \cdot n_2$ states. □

As has been the case with DBW-recognizability, one can generate certificates from a DBW-sep-refuter. The proof is similar to that of Theorem 3, with membership in $L_1$ replacing membership in $L$ and membership in $L_2$ replacing being disjoint from $L$. Formally, we have the following.

**Theorem 7.** *Two $\omega$-regular languages $L_1, L_2 \subseteq \Sigma^\omega$ are not DBW-separable iff there exist three finite words $x \in \Sigma^*$ and $x_1, x_2 \in \Sigma^+$, such that $x \cdot (x_1 + x_2)^* \cdot x_1^\omega \subseteq L_1$ and $x \cdot (x_1^* \cdot x_2)^\omega \subseteq L_2$.*

We refer to a triple $\langle x, x_1, x_2\rangle$ of words that satisfy the conditions in Theorem 7 as a *certificate* to the non-DBW-separability of $\langle L_1, L_2\rangle$. Observe that the same way we generated a no-DBW witness in Remark 3, we can extract, given a DBW-sep-refuter $\mathcal{R}$ for $\langle L_1, L_2\rangle$, languages $L_1' \subseteq L_1$ and $L_2' \subseteq L_2$ that tighten $\langle L_1, L_2\rangle$ and are still not DBW-separable.

### 4.2 Certificate-Guided Approximation

In this section we describe a method for finding small approximating languages $I_\downarrow$ and $I_\uparrow$ such that $\langle L \setminus I_\downarrow, \mathit{comp}(L) \setminus I_\uparrow\rangle$ is DBW-separable. If this method terminates we obtain an approximation for $L$ that is DBW-recognizable. As in *counterexample guided abstraction-refinement* (CEGAR) for model checking [10], we use certificates for non-DBW-separability in order to suggest interesting approximating languages. Intuitively, while in CEGAR the refined system excludes the counterexample, here the approximation of $L$ excludes the certificate.

Consider a certificate $\langle x, x_1, x_2\rangle$ for the non-DBW-separability of $\langle L_1, L_2\rangle$. We suggest the following five approximations:

$$\begin{array}{ll}
C_0 = x \cdot (x_1 + x_2)^\omega & \rightsquigarrow \langle L_1 \setminus C_0, L_2 \setminus C_0\rangle \\
C_1 = x \cdot (x_1 + x_2)^* \cdot x_1^\omega = L_1 \cap C_0 & \rightsquigarrow \langle L_1 \setminus C_1, L_2\rangle \\
C_2 = x \cdot (x_2^* \cdot x_1)^\omega \supset C_1 & \rightsquigarrow \langle L_1, L_2 \setminus C_2\rangle \\
C_3 = x \cdot (x_1^* \cdot x_2)^\omega = L_2 \cap C_0 & \rightsquigarrow \langle L_1, L_2 \setminus C_3\rangle \\
C_4 = x \cdot (x_1 + x_2)^* \cdot x_2^\omega \subset C_3 & \rightsquigarrow \langle L_1, L_2 \setminus C_4\rangle
\end{array}$$

First, it is easy to verify that $\langle x, x_1, x_2\rangle$ is indeed not a certificate for the non-DBW-separability of the obtained candidate pairs $\langle L_1', L_2'\rangle$. If $\langle L_1', L_2'\rangle$ is DBW-separable, we are done (yet may try to tighten the approximation). Otherwise, we can repeat the process with a certificate for the non-DBW-separability of $\langle L_1', L_2'\rangle$. As in CEGAR, some suggestions may be more interesting than others, in some cases the process terminates, in some it does not, and the user takes part directing the search.

*Example 6.* Consider again the language $L = (a + b)^* \cdot b^\omega$ and the certificate $\langle x, x_1, x_2\rangle = \langle \epsilon, b, a\rangle$. Trying to approximate $L$ by a language in DBW, we start with the pair $\langle L, \mathit{comp}(L)\rangle$. Our five suggestions are then as follows.

$$\begin{array}{ll}
C_0 = \Sigma^\omega & \rightsquigarrow \langle L \setminus C_0, \mathit{comp}(L) \setminus C_0\rangle = \langle \emptyset, \emptyset\rangle \\
C_1 = (b + a)^* \cdot b^\omega & \rightsquigarrow \langle L \setminus C_1, \mathit{comp}(L)\rangle = \langle \emptyset, \mathit{comp}(L)\rangle \\
C_2 = (a^* \cdot b)^\omega & \rightsquigarrow \langle L, \mathit{comp}(L) \setminus C_2\rangle = \langle L, (a + b)^* \cdot a^\omega\rangle \\
C_3 = (b^* \cdot a)^\omega & \rightsquigarrow \langle L, \mathit{comp}(L) \setminus C_3\rangle = \langle L, \emptyset\rangle \\
C_4 = (b + a)^* \cdot a^\omega & \rightsquigarrow \langle L, \mathit{comp}(L) \setminus C_4\rangle = \langle L, (a + b)^* \cdot (a \cdot a^* \cdot b \cdot b^*)^\omega\rangle
\end{array}$$

Candidates $C_0$, $C_1$, and $C_3$ induce trivial approximations. Then, $C_2$ suggests to over-approximate $L$ by setting $I_\uparrow$ to $(a^* \cdot b)^\omega$, which we view as a nice solution, approximating "eventually always $b$" by "infinitely often $b$". Then, the pair derived from $C_4$ is not DBW-separable. We can try to approximate it. Note, however, that repeated approximations in the spirit of $C_4$ are going to only extend the prefix of $x$ in the certificates, and the process does not terminate. In the full version of this article [27], we describe the process for the certificate $\langle x, x_1, x_2 \rangle = \langle a, b, a \rangle$, which again might not terminate. ■

## 5 Other Classes of Deterministic Automata

In this section we generalise the idea of DBW-refuters to other classes of deterministic automata. For this we take again the view that a deterministic automaton is a $\langle \Sigma, A \rangle$-transducer over a suitable annotation alphabet $A$. We then characterize each class of deterministic automata by two languages over $A$:

– The language $L_{\text{acc}} \subseteq A^\omega$, describing when a run is accepting. For example, for DBWs, we have $A = \{\text{ACC}, \text{REJ}\}$ and $L_{\text{acc}} = \infty\text{ACC}$.
– The language $L_{\text{struct}} \subseteq A^\omega$, describing structural conditions on the run. For example, recall that a DWW is a DBW in which the states of each SCS are either all accepting or all rejecting, and so each run eventually get trapped in an accepting or rejecting SCS. Accordingly, the language of runs that satisfy the structural condition is $L_{\text{struct}} = A^* \cdot (\text{ACC}^\omega + \text{REJ}^\omega)$.

We now formalize this intuition. Let $A$ be a finite set of annotations and let $\gamma = \langle L_{\text{acc}}, L_{\text{struct}} \rangle$, for $L_{\text{acc}}, L_{\text{struct}} \subseteq A^\omega$. A deterministic automaton $\mathcal{A} = \langle \Sigma, Q, q_0, \delta, \alpha \rangle$ is a deterministic $\gamma$ automaton (D$\gamma$W, for short) if there is a function $\tau \colon Q \to A$ that maps each state to an annotation such that a run $r$ of $\mathcal{A}$ satisfies $\alpha$ iff $\tau(r) \in L_{\text{acc}}$, and all runs $r$ satisfy the structural condition, thus $\tau(r) \in L_{\text{struct}}$. We then say that a language $L$ is $\gamma$-recognizable if there a D$\gamma$W $\mathcal{A}$ such that $L = L(\mathcal{A})$.

Before we continue to study $\gamma$-recognizability, let us demonstrate the $\gamma$-characterization of common deterministic automata. We first start with classes $\gamma$ for which $L_{\text{struct}}$ is trivial; i.e., $L_{\text{struct}} = A^\omega$.

– DBW: $A = \{\text{ACC}, \text{REJ}\}$ and $L_{\text{acc}} = \infty\text{ACC}$.
– DCW: $A = \{\text{ACC}, \text{REJ}\}$ and $L_{\text{acc}} = \neg\infty\text{ACC}$.
– DPW$[i, k]$: $A = \{i, \ldots, k\}$ and $L_{\text{acc}} = \{y \in A^\omega : \max(\mathit{inf}(y)) \text{ is odd}\}$.
– DELW$[\theta]$: $A = 2^{\mathbb{M}}$ and $L_{\text{acc}} = \{y \in A^\omega : y \models \theta\}$.

Note that the characterizations for Büchi, co-Büchi, and parity are special cases of the characterization for DELW. In a similar way, we could define a language $L_{\text{acc}}$ for DRW$[k]$ and other common special cases of DELWs. We continue to classes in the depth hierarchy, where $\gamma$ includes also a structural restriction:

– DWW: The set $A$ and the language $L_{\text{acc}}$ are as for DBW or DCW. In addition, $L_{\text{struct}} = A^* \cdot (\text{ACC}^\omega + \text{REJ}^\omega)$.
– DWW$[j, k]$, for $j \in \{0, 1\}$: The set $A$ and the language $L_{\text{acc}}$ are as for DPW$[j, k]$. In addition, $L_{\text{struct}} = \{y_0 \cdot y_1 \cdots \in A^\omega : \text{for all } i \geq 0, \text{ we have that } y_i \leq y_{i+1}\}$.

- *Bounded Languages*: A language $L$ is bounded if it is both safety and co-safety. Thus, every word $w \in \Sigma^\omega$ has a prefix $v \in \Sigma^*$ such that either for all $u \in \Sigma^\omega$ we have $v \cdot u \in L$, or for all $u \in \Sigma^\omega$ we have $v \cdot u \notin L$ [23]. To capture this, we use $A = \{\textsc{acc}, \textsc{rej}, ?\}$, where "?" is used for annotating states with both accepting and rejecting continuations. Then, $L_{\text{acc}} = A^* \cdot \textsc{acc}^\omega$, and $L_{\text{struct}} = ?^* \cdot (\textsc{acc}^\omega + \textsc{rej}^\omega)$.
- *Deterministic* $(m, n)$*-Superparity Automata* [39]: $A = \{(i, j) : 0 \leq i \leq m, 0 \leq j \leq n\}$, $L_{\text{acc}} = \{y_m \oplus y_n \in A^\omega : \max(\mathit{inf}(y_m)) + \max(y_n) \text{ is odd}\}$, and $L_{\text{struct}} = \{y_m \oplus (y_0 \cdot y_1 \cdots) \in A^\omega : y_i \leq y_{i+1}, \text{ for all } i \geq 0\}$.

Let $\Sigma$ be an alphabet, let $A$ be an annotation alphabet, and let $\gamma = \langle L_{\text{acc}}, L_{\text{struct}}\rangle$, for $L_{\text{acc}}, L_{\text{struct}} \subseteq A^\omega$. We define the language $\text{Real}(L, \gamma) \subseteq (\Sigma \times A)^\omega$ of words with correct annotations.

$$\text{Real}(L, \gamma) = \{x \oplus y : y \in L_{\text{struct}} \text{ and } (x \in L \text{ iff } y \in L_{\text{acc}})\}.$$

Note that the language $\text{DBW}(L)$ can be viewed as a special case of our general framework. In particular, in cases $L_{\text{struct}} = A^\omega$, we can remove the $y \in L_{\text{struct}}$ conjunct from $\text{Real}(L, \gamma)$. Note that $\mathit{comp}(\text{Real}(L, \gamma))$ is the language

$$\text{NoReal}(L, \gamma) = \{x \oplus y : y \notin L_{\text{struct}} \text{ or } (x \in L \text{ iff } y \notin L_{\text{acc}})\}.$$

A $\gamma$*-refuter for* $L$ is then an $(A/\Sigma)$-transducer with $\iota = \mathit{env}$ that realizes $\text{NoReal}(L, \gamma)$. We can now state the "D$\gamma$W-generalization" of Proposition 2.

**Proposition 4.** *Consider an* $\omega$*-regular language* $L \subseteq \Sigma^\omega$*, and a pair* $\gamma = \langle L_{\text{acc}}, L_{\text{struct}}\rangle$*, for* $\omega$*-regular languages* $L_{\text{acc}}, L_{\text{struct}} \subseteq A^\omega$*. Exactly one of the following holds:*

1. $L$ *is in* D$\gamma$W*, in which case the language* $\text{Real}(L, \gamma)$ *is* $(\Sigma/A)$*-realizable by the system, and a finite-memory winning strategy for the system induces a* D$\gamma$W *for* $L$*.*
2. $L$ *is not in* D$\gamma$W*, in which case the language* $\text{NoReal}(L, \gamma)$ *is* $(A/\Sigma)$*-realizable by the environment, and a finite-memory winning strategy for the environment induces a* $\gamma$*-refuter for* $L$*.*

Note that every DELW can be complemented by dualization, thus by changing its acceptance condition from $\theta$ to $\neg\theta$. In particular, DBW and DCW dualize each other. As we argue below, dualization is carried over to refutation. For example, the $(\{\textsc{acc}, \textsc{rej}\}/\Sigma)$-transducer $\mathcal{R}$ from Figure 1 is both a DBW-refuter for $\neg\infty a$ and a DCW-refuter for $\infty a$. Formally, we have the following.

**Theorem 8.** *Consider an EL-condition* $\theta$ *over* $\mathbb{M}$*. Let* $A = 2^{\mathbb{M}}$*. For every* $(A/\Sigma)$*-transducer* $\mathcal{R}$ *and language* $L$*, we have that* $\mathcal{R}$ *is a* DELW$[\theta]$*-refuter for* $L$ *iff* $\mathcal{R}$ *is a* DELW$[\neg\theta]$*-refuter for* $\mathit{comp}(L)$*. In particular, for every language* $L$ *and* $(\{\textsc{acc}, \textsc{rej}\}/\Sigma)$*-transducer* $\mathcal{R}$*, we have that* $\mathcal{R}$ *is a DBW-refuter for* $L$ *iff* $\mathcal{R}$ *is a DCW-refuter for* $\mathit{comp}(L)$*.*

*Proof.* For DELW[$\theta$]-recognizability of $L$, the language of correct annotations is $\{x \oplus y : (x \in L \text{ iff } y \models \theta)\}$, which is equal to $\{x \oplus y : (x \in comp(L) \text{ iff } y \models \neg\theta)\}$, which is the language of correct annotations for DELW[$\neg\theta$]-recognizability of $comp(L)$. □

While dualization is nicely carried over to refutation, this is not the case for all expressiveness results. For example, while DWW=DBW∩DCW, and in fact DBW and DCW are weak type (that is, when the language of a DBW is in DWW, an equivalent DWW can be defined on top of its structure, and similarly for DCW [21]), we describe in [27] a DWW-refuter that is neither a DBW- nor a DCW-refuter. Intuitively, this is possible as in DWW refutation, Prover loses when the input is not in $A^* \cdot (\textsc{acc}^\omega + \textsc{rej}^\omega)$, whereas in DBW and DCW refutation, Refuter has to respond correctly also for these inputs.

On the other hand, as every DWW is also a DBW and a DCW, every DBW-refuter or DCW-refuter is also a DWW-refuter.

It is easy to see that our results about D$\gamma$W-recognizability can be extended to separability and approximation in the same way DBW-recognizability has been extended in Section 4. We describe the details in the full version [27], as well as word-certificates for the non-D$\gamma$W-recognizability and -separability of several well-known types of $\gamma$.

## 6 Discussion and Directions for Future Research

The automation of decision procedures makes certification essential. We suggest to use the winning strategy of the refuter in expressiveness games as a certificate to inexpressibility. We show that beyond this *state-based certificate*, the strategy induces a *word-based certificate*, generated from words traversed along a "flower structure" the strategy contains, as well as a *language-based certificate*, consisting of languages that under- and over-approximate the language in question and that are not separable by automata in the desired class.

While our work considers *expressive power*, one can use similar ideas in order to question the *size* of automata needed to recognize a given language. For example, in the case of a regular language $L$ of finite words, the Myhill-Nerode characterization [37,38] suggests to refute the existence of deterministic finite word automata (DFW) with $n$ states for $L$ by providing $n + 1$ prefixes that are not right-congruent. Using our approach, one can alternatively consider the winning strategy of Refuter in a game in which the set of annotations includes also the state space, and $L_{\text{struct}}$ ensures consistency of the transition relation. Even more interesting is refutation of size in the setting of automata on infinite words. Indeed, there, minimization is NP-complete [46], and there are interesting connections between polynomial certificates and possible membership in co-NP, as well as connections between size of certificates and succinctness of the different classes of automata.

Finally, while the approximation scheme we studied is based on suggested over- and under-approximating languages, it is interesting to study approximations that are based on more flexible distance measures [13,18].
